# Global burden of cancer attributable to HIV: a worldwide incidence analysis

**DOI:** 10.1016/S2214-109X(25)00264-5

**Published:** 2025-08-19

**Authors:** Yue Huang, Damien Georges, Harriet Rumgay, Isabelle Soerjomataram, Gary M Clifford

**Affiliations:** a Early Detection, Prevention and Infections Branch, International Agency for Research on Cancer (IARC/WHO), Lyon, France; b Cancer Surveillance Branch, International Agency for Research on Cancer (IARC/WHO), Lyon, France

## Abstract

**Background:**

People living with HIV are at increased risk of multiple infection-related cancers due to HIV-driven immunosuppression. However, the global burden of cancer attributable to HIV remains unestimated. We aimed to comprehensively estimate the global and regional cancer burden attributable to HIV in 2022, and to understand geographical disparities in this burden.

**Methods:**

Nine cancer types considered causally linked to HIV infection were assessed in this worldwide incidence analysis: Kaposi sarcoma; non-Hodgkin lymphoma; Hodgkin lymphoma; and cervical, anal, penile, vulvar, vaginal, and conjunctival cancer. Cancer-specific population attributable fractions (PAFs) were calculated, primarily from meta-analyses reporting relative risks and HIV prevalence estimates sourced from UNAIDS 2023. PAFs were applied to national cancer incidence estimates from GLOBOCAN 2022 to estimate the numbers and age-standardised incidence rates (ASIRs) of HIV-attributable cancers for 185 countries and territories.

**Findings:**

In 2022, 0·4% of global cancer cases (81 300 of 19 million) were estimated to be attributable to HIV, largely driven by cervical cancer (n=30 500, HIV-attributable cancer-specific PAF 4·6%), Kaposi sarcoma (n=24 500, 70·6%), and non-Hodgkin lymphoma (n=12 800, 2·4%). 57 300 (70·5%) of the 81 300 HIV-attributable cancer cases occurred in Africa, particularly in eastern (33 800 [41·6%] cases) and southern Africa (14 000 [17·2%] cases), where HIV was the cause of more than 10% of all cancers. ASIRs of HIV-attributable cancer were lowest in Asia (0·2 per 100 000) and reached 27·6 per 100 000 in southern Africa. The relative importance of HIV-attributable cancers varied globally, with cervical cancer accounting for 40·8% (23 400 of 57 300 cases) of HIV-attributable cancer in Africa, but less than 10% in North America (200 [5·0%] of 4000 cases) and in northern and western Europe (100 [5·3%] of 1900 cases), where anal cancer (900 [22·5%] and 500 [26·3%] cases), Kaposi sarcoma (900 [22·5%] and 500 [26·3%] cases), and non-Hodgkin lymphoma (1400 [35·0%] and 500 [26·3%] cases) were more common.

**Interpretation:**

The absolute and relative HIV-attributable cancer burden varies globally. These data can inform region-specific planning and evaluation of HIV control, as well as cancer-specific interventions such as vaccination and screening, to reduce the infection-related cancer burden in people living with HIV.

**Funding:**

None.

## Introduction

The number of people living with HIV is increasing globally, rising from 27·2 million in 2000 to 39·9 million in 2023, despite reductions in the annual number of new HIV infections.^1^ This increase is due to wider and earlier access to antiretroviral therapy (ART), which has substantially increased life expectancy among people living with HIV. However, as this population ages, morbidity and mortality from non-communicable diseases, including cancer, are becoming increasingly important.

People living with HIV have a considerably higher risk of multiple types of infection-related cancers compared with the general population.^2^ HIV indirectly causes cancer through immunosuppression, worsening the natural history of other carcinogenic viruses, most notably human herpesvirus type 8 (HHV-8) for Kaposi sarcoma, human papillomavirus (HPV) for anogenital cancers, and Epstein–Barr virus (EBV) for non-Hodgkin lymphoma and Hodgkin lymphoma. The International Agency for Research on Cancer (IARC) first classified HIV as carcinogenic to humans in 1996,^3^ and its assessment in 2012 deemed there to be sufficient evidence of a causal link between HIV and Kaposi sarcoma, non-Hodgkin lymphoma, Hodgkin lymphoma, cervical cancer, squamous cell carcinoma of the anus, and squamous cell carcinoma of the conjunctiva.^2^ All of these cancers, except for conjunctival cancer, have an established underlying infectious cause. Since then, evidence has also accumulated for a causal excess risk of cancers of the vulva, vagina, and penis among people living with HIV,^4^, ^5^ through a pathway of HPV carcinogenesis analogous to cervical and anal cancers.

**Research in context**

**Evidence before this study**

We searched PubMed using the keywords (“Human Immunodeficiency Virus” OR “HIV”) AND (“cancer” OR “neoplasm” OR “carcinoma”) AND “attribut*”, to identify publications on national and global estimates of the cancer burden attributable to HIV. Previous studies have reported the global HIV-attributable burden of five cancer sites separately: namely, cervical cancer, anal cancer, Kaposi sarcoma, non-Hodgkin lymphoma, and conjunctival cancer. But no study has reported the global burden of these cancers combined, nor included the global burden of other HIV-attributable cancer sites for which the growing evidence also suggests a causal link—namely, cancers of the vulva, vagina, and penis, as well as Hodgkin lymphoma. At the individual country level, studies have estimated that 0·50% of cancer cases in the USA, 1·20% in Italy, and 0·03% in South Korea were attributable to HIV, but these studies did not include all the above cancer sites now recognised to be attributable to HIV.

**Added value of this study**

This analysis is, to the best of our knowledge, the first comprehensive assessment of the global burden of HIV-attributable cancer. We estimated that 0·4% of global cancer cases (81 300 of 19 million) diagnosed in 2022 were attributable to HIV and could be theoretically preventable by improved HIV control measures. Globally, cervical cancer bears the largest share of cancers attributable to HIV, followed by Kaposi sarcoma, non-Hodgkin lymphoma, and Hodgkin lymphoma. We also highlighted considerable geographical disparities in the HIV-attributable cancer burden in absolute and relative terms, noting that the largest HIV-attributable cancer burden was found in sub-Saharan Africa, particularly in eastern and southern Africa, where HIV contributed more than 10% of all cancer cases. The types of cancers attributable to HIV varied widely across different world regions, with cervical cancer accounting for 41·0% of all HIV-attributable cancers in Africa, while anal cancer and non-Hodgkin lymphoma contributed the majority of HIV-attributable cancers (>50%) in North America and western Europe.

**Implications of all the available evidence**

The combined burden of nine cancer types considered attributable to HIV can inform national plans for HIV control and its potential impact on infection-related cancer burden in people living with HIV. It can also inform on priorities for interventions aimed to reduce the cancer burden such as human papillomavirus vaccination as well as cervical and anal cancer screening among people living with HIV in different world regions. These priorities are underpinned by considerable geographical disparities, with a pronounced concentration of HIV-attributable cervical cancer and Kaposi sarcoma in sub-Saharan Africa, and a relatively greater burden of anal cancer and non-Hodgkin lymphoma in high-income settings.

By expanding on previous methods specifically developed for Kaposi sarcoma,^6^ cervical cancer,^7^ anal cancer,^8^ and conjunctival cancer,^9^ we aimed to comprehensively estimate the global and regional cancer burden attributable to HIV in 2022, and to understand geographical disparities in this burden. Ultimately, our aim was to provide the best current estimate to inform HIV control programmes and targeted interventions to reduce the infection-related cancer burden in people living with HIV.

## Methods

### Definition of cancer sites attributable to HIV

In 2012, an IARC expert working group concluded that there was sufficient evidence for a causal association of HIV infection with Kaposi sarcoma, non-Hodgkin lymphoma, Hodgkin lymphoma, cervical cancer, squamous cell carcinoma of the anus (hereafter referred to as anal cancer), and squamous cell carcinoma of the conjunctiva (hereafter referred to as conjunctival cancer).^2^ Since then, evidence has accumulated of considerable relative risks (RRs) of HPV-related cancers of the vulva, vagina, and penis in people living with HIV,^4^, ^5^ which are similar to those of HPV-related cervical and anal cancer, and cannot be explained by known confounding factors. Thus, we considered the above nine cancer sites to be causally related to HIV.

The incidence of other cancer sites has been reported to be considerably elevated in people living with HIV in some studies, but these sites were not included in our analyses due to the absence of consensus on possible confounding (eg, lung or upper digestive tract cancers and smoking), or a paucity of appropriate methodology and data available (eg, liver cancer related to hepatitis B virus [HBV] and hepatitis C virus [HCV]).

### Relative risk estimates

Estimates of RRs were derived from comparisons of cancer incidence in people living with HIV versus HIV-negative individuals or versus the general population, stratified by age, sex, or other key determinants, as relevant (appendix p 7). For cervical,^7^ anal,^8^ and conjunctival cancer,^9^ we obtained RRs from previous studies estimating population attributable fractions (PAFs) for these cancers (appendix p 7). For cancers of the vulva, vagina, and penis, and Hodgkin lymphoma, we obtained RRs from a recent meta-analysis of cancer risks in people living with HIV,^5^ irrespective of age. For non-Hodgkin lymphoma, age-specific RRs for non-Hodgkin lymphoma in the most recently available period (2013–19) were obtained from the US HIV/AIDS Cancer Match Study.^10^

### HIV prevalence

We sourced HIV prevalence estimates for individuals aged 15 years and older from UNAIDS 2023^11^ for 178 of 185 countries and territories in GLOBOCAN 2022 (a description of GLOBOCAN 2022 is provided below). For the remaining seven GLOBOCAN countries and territories (Samoa, Saint Lucia, Puerto Rico, Vanuatu, Guam, West Bank and Gaza Strip, and Solomon Islands) not included in the UNAIDS dataset, we obtained HIV prevalence data from the 2021 Global Burden of Diseases, Injuries, and Risk Factors Study (GBD).

### Cancer burden

GLOBOCAN 2022 provides the number of cases for 36 major cancer sites across 185 countries and territories, categorised by sex and age. For all cancers except for conjunctival cancer, burden estimates in the population aged 15 years and older were available directly from GLOBOCAN 2022, including Kaposi sarcoma (C46, International Classification of Diseases [ICD], 10th revision, version 2010); non-Hodgkin lymphoma (C82–86, C88); Hodgkin lymphoma (C81); cancers of the cervix (C53), anus (C21), penis (C60), vulva (C51), and vagina (C52); and all cancers combined excluding non-melanoma skin cancer (C00–97 excluding C44). We extracted proportions of squamous cell carcinoma within total microscopically verified anal cancer cases from CI5 X–XII registry data, supplemented by updated data from the African Cancer Registry Network, as previously reported,^8^, ^12^ and numbers of anal squamous cell carcinoma cases were estimated by applying these proportions to the estimated numbers of anal cancer cases in GLOBOCAN. Since estimates of conjunctival cancer were not available in GLOBOCAN 2022, we estimated cases using previously reported rates for countries in Africa.^9^ Conjunctival cancer outside Africa was not considered, due to the small number (<50) of these cases.

### Statistical analysis

We assumed the prevalence of HIV infection in Kaposi sarcoma cases equates to the PAF, due to the very high RR for Kaposi sarcoma in people living with HIV, as described previously.^6^ For all other cancer sites, we estimated the cancer-specific PAF (equation 1) and proportion of cancer cases in people living with HIV (equation 2) from the RR and the prevalence of HIV in the population, according to sex, age, geographical subgroups, or a combination of the above (appendix p 7), as previously reported for cervical,^7^ anal,^8^ and conjunctival cancer.^9^

$$\begin{array}{l} \text{PAF}=\frac{\text{HIV}\;\text{prevalence}\times \left(\text{RR}-1\right)}{1+\text{HIV}\;\text{prevalence}\times \left(\text{RR}-1\right)}\\ \text{Proportion}=\frac{\text{HIV}\;\text{prevalence}\times \text{RR}}{\left(1-\text{HIV}\;\text{prevalence}\right)+\text{HIV}\;\text{prevalence}\times \text{RR}} \end{array}$$

Multiplying the number of cancer cases estimated in GLOBOCAN by the PAF or proportion of cancer cases in people living with HIV yielded estimates for the total number of new cancer cases attributable to HIV or cancer cases in people living with HIV, by cancer type, country, and sex. We then estimated the proportion of HIV-attributable cancer cases among all cancer cases (PAF_all cancer_) by dividing the overall number of HIV-attributable cancer cases by the total number of all cancer cases.

Additionally, we calculated age-standardised incidence rates (ASIRs) of cancer attributable to HIV per 100 000 person-years in 2022. The standard world population used was Segi (1960) modified by Doll (1966), as described in the Cancer Incidence in Five Continents website.^13^ We aggregated country-specific estimates worldwide according to UN regions and subregions. All statistical analyses were conducted in R (version 4.3.2).

### Role of the funding source

There was no funding source for this study.

## Results

**Figure 1:**
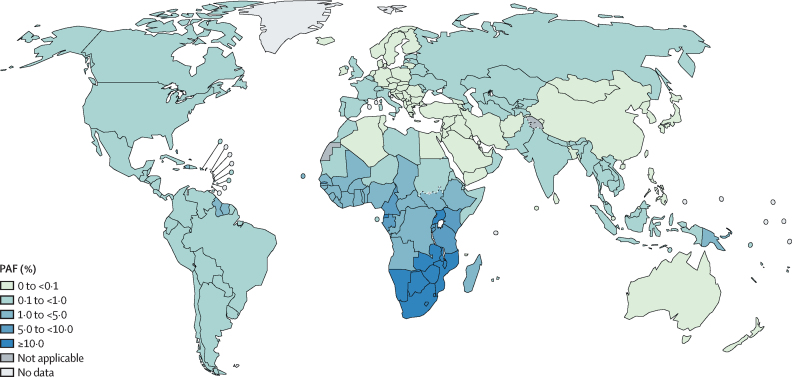
Overall population attributable fractions of cancer cases attributable to HIV by country in 2022 Cancer cases attributable to HIV by countries are provided in the appendix (pp 9–15). PAF=population attributable fraction.

Globally, 81 300 of 19 million cancer cases diagnosed in 2022 were estimated to be attributable to HIV infection, accounting for approximately 0·4% of all cancer cases (table 1). The proportion of all cancer cases attributable to HIV varied substantially worldwide, from 0·1% in Asia to more than 10% in eastern and southern Africa (table 1). PAFs ranged from less than 0·1% in 64 countries (mostly in western Asia, northern Europe, eastern Asia, as well as Australia and New Zealand) to more than 10% in 11 countries, all located in sub-Saharan Africa (figure 1, sex-specific PAFs showed in the appendix pp 2–3).

**Table 1:** Estimated burden of cancers attributable to HIV in the population aged 15 years and older in 2022

		Number of all cancer cases*, 2022	**Cancer cases attributable to HIV, 2022**		
			Number	ASIR (per 100 000)	PAF†
Global		18 541 200	81 300	1·5	0·4%
Sex					
	Female	9 088 200	50 000	1·8	0·6%
	Male	9 453 000	31 300	1·1	0·3%
World region‡					
	Africa	1 106 700	57 300	7·7	5·2%
	Asia	9 604 500	7600	0·2	0·1%
	Southern, central, and eastern Europe	2 218 500	4900	1·2	0·2%
	Western and northern Europe	1 906 000	1900	0·6	0·1%
	Latin America and the Caribbean	1 456 600	5300	1·0	0·4%
	North America	2 053 400	4000	1·2	0·2%
	Oceania	195 500	300	0·9	0·2%
Africa subregion‡					
	Eastern Africa	324 100	33 800	14·4	10·4%
	Middle Africa	106 700	3800	4·3	3·6%
	Northern Africa	322 200	200	0·1	0·1%
	Southern Africa	107 700	14 000	27·6	13·0%
	Western Africa	246 000	5500	2·8	2·2%

ASIR=age-standardised incidence rate. PAF=population attributable fraction.

* Non-melanoma skin cancer was excluded.

† Overall PAFs in all cancer cases.

‡ World regions and Africa subregions were grouped according to UN definitions.

**Figure 2:**
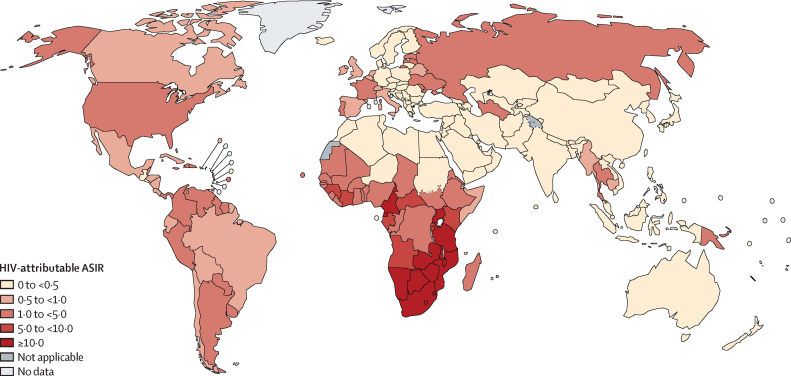
Age-standardised incidence rates of cancer cases attributable to HIV by country in 2022 ASIRs of cancer cases attributable to HIV by countries are provided in the appendix (pp 9–15). ASIR=age-standardised incidence rate.

The global ASIR for HIV-attributable cancer cases was 1·5 cases per 100 000 person-years and was higher in women (1·8 cases per 100 000) than in men (1·1 cases per 100 000; table 1). ASIRs for HIV-attributable cancer varied greatly worldwide, from 0·2 per 100 000 person-years in Asia to 7·7 cases per 100 000 person-years in Africa, reaching 27·6 cases per 100 000 person-years in southern Africa (table 1). ASIRs ranged from less than 0·1 cases per 100 000 in 30 countries to more than 40 cases per 100 000 person-years in Eswatini, Zimbabwe, and Malawi (figure 2; appendix pp 9–15). Sex-specific ASIRs are shown in the appendix (pp 4–5).

**Figure 3:**
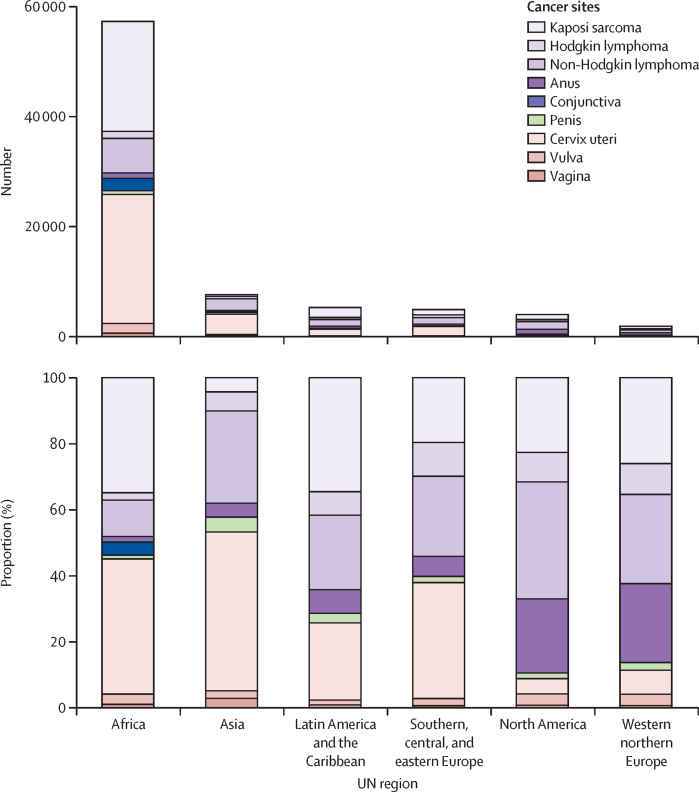
Absolute and relative numbers of cancer cases attributable to HIV across different UN regions or subregions The cancer cases attributable to HIV in Oceania are not shown here due to small numbers.

The global HIV-attributable cancer burden included 30 500 cases of cervical cancer, 24 500 cases of Kaposi sarcoma, 12 800 cases of non-Hodgkin lymphoma, and 3400 cases of anal cancer, along with 10 100 cases combined of Hodgkin lymphoma, conjunctival cancer, and penile, vulval, and vaginal cancers (table 2). However, the relative frequency of these cancers varied by region (figure 3). Global HIV PAFs ranged from 2·4% for non-Hodgkin lymphoma to 70·6% for Kaposi sarcoma (table 2). The equivalent numbers of specific cancer cases in people living with HIV included 37 400 cervical cancers (5·7% of the global burden of cervical cancer), 24 500 (70·6%) Kaposi sarcoma cases, 14 800 (2·8%) non-Hodgkin lymphoma cases, and 3500 (10·8%) anal cancer cases (table 2).

**Table 2:** Estimated number of HIV-attributable cancer cases globally in 2022, by cancer site*

	**Number of cancer cases, 2022**	**Cases attributable to HIV, 2022**		Cases in people living with HIV*, 2022	
		Number	Cancer-specific PAF†	Number	Cancer-specific Proportion
Cervix uteri (C53)	662 200	30 500	4·6%	37 400	5·7%
Kaposi sarcoma (C46)	34 700	24 500	70·6%	24 500	70·6%
Non-Hodgkin lymphoma (C82–86, C88)	535 800	12 800	2·4%	14 800	2·8%
Squamous cell carcinoma of the anus (C21)‡	32 400	3400	10·5%	3500	10·8%
Hodgkin lymphoma (C81)	74 600	3100	4·2%	3500	4·7%
Vulva (C51)	47 200	2300	4·9%	2500	5·3%
Squamous cell carcinoma of the conjunctiva (C69)‡	6700	2300	34·3%	2500	37·3%
Penis (C60)	37 600	1400	3·7%	1600	4·3%
Vagina (C52)	18 700	1000	5·3%	1000	5·3%

PAF=population attributable fraction. ICD-10=International Classification of Diseases, 10th revision. Cases are ordered by the number attributable to HIV.

* Irrespective of being attributable to HIV or not.

† PAFs in the specific cancer.

‡ Squamous cell carcinoma of the anus (ICD-10: C21, ICD-O-3: 8050–76, 8083–84, 8123–24); squamous cell carcinoma of the conjunctiva (ICD-10: C69, ICD-O-3: 8050–78, 8083–84).

Cancers attributable to HIV were diagnosed predominantly in Africa (57 300 cases) accounting for 70·5% of the global HIV-attributable burden (figure 3, table 1). The relative contribution of specific cancers to the overall HIV-attributable cancer burden varied by world region (figure 3; the sex-specific pattern in shown in the appendix p 6). Contrary to the patterns seen in Africa and Asia, where a higher burden was observed in women than in men, in the Americas and western Europe the burden was greater among men than among women. The high HIV-attributable cancer burden in Africa was driven particularly by cervical cancer, which accounted for 40·8% (23 400 of 57 300 cases) of the HIV-attributable cancer burden in this region, and Kaposi sarcoma, which accounted for 34·9% (20 000 cases; appendix p 16). By contrast, in western and northern Europe and North America, cervical cancer represented a relatively small proportion of HIV-attributable cancer cases (100 [5·3%] of 1900 cases in western and northern Europe and 200 [5·0%] of 4000 cases in North America), while the proportions of anal cancer (900 [22·5%] cases in North America and 500 [26·3%] cases in northern and western Europe), non-Hodgkin lymphoma (1400 [35·0%] cases in North America and 500 [26·3%] cases in northern and western Europe), and Kaposi sarcoma (900 [22·5%] cases in North America and 500 [26·3%] cases in northern and western Europe) were relatively high. The contribution of Kaposi sarcoma to the overall HIV-attributable cancer burden was notably low in Asia (300 [3·9%] of 7600 cases).

## Discussion

In this comprehensive summary of the global burden of HIV-attributable cancer, an estimated 0·4% (81 300 of 19 million) of cancer cases diagnosed in 2022 could be attributed to HIV and hence were theoretically preventable by improving HIV control measures. The burden varied considerably by region, with sub-Saharan Africa carrying by far the largest HIV-attributable cancer burden, in terms of highest PAFs, highest absolute numbers, and highest ASIRs. Countries in eastern and southern Africa were most affected, with more than 10% of all cancer cases estimated to be attributable to HIV infection, driven by the high population prevalence of HIV in the region.^11^ Furthermore, this work highlights how the distribution of the four most common HIV-attributable cancers—Kaposi sarcoma, non-Hodgkin lymphoma, and cervical and anal cancer—varies by region, driven by differences in access to preventive services and in the concentration of the HIV epidemic in men who have sex with men (MSM).

Globally, cervical cancer constitutes the largest share of cancers attributable to HIV, accounting for nearly half of the burden in Africa and Asia. Although the global cancer-specific PAF for cervical cancer was relatively low (4·6%), cervical cancer PAFs exceed 50% in certain countries in sub-Saharan Africa^7^ that have the highest prevalence of HIV and cervical cancer. Specific guidelines for women living with HIV have been issued by WHO for cervical cancer screening^14^ and HPV vaccination,^15^ which is expected to additionally prevent HPV-attributable cancer at other anogenital sites. In high-burden settings, modelling studies have shown that tailored interventions through a combination of scaling up ART, HPV vaccination, and cervical cancer screening can achieve long-term reductions in cervical cancer incidence among women living with HIV.^16^ Cervical cancer contributed a relatively small proportion of HIV-attributable cancer in North America and western and northern Europe, which is likely to be due to widespread access to cervical cancer screening, and a relatively higher concentration of the HIV epidemic in men (especially MSM).

Kaposi sarcoma remained the second most diagnosed HIV-attributable cancer globally, despite being highly preventable through avoidance of severe immunosuppression via early access to ART.^17^ As shown previously,^6^ the majority of the HIV-attributable Kaposi sarcoma burden was found in sub-Saharan Africa, where the reduction in incidence of Kaposi sarcoma among people living with HIV has not been as pronounced as in high-income regions, probably due to suboptimal ART access.^18^ This highlights HIV control and treatment as the most important approach to reducing the prevalence of Kaposi sarcoma, particularly in Africa, given the current paucity of effective interventions for HHV-8 primary prevention.^17^

Non-Hodgkin lymphoma was the predominant HIV-attributable cancer in North America and in western and northern Europe. The cancer-specific attributable fraction for non-Hodgkin lymphoma (2·4%) was the smallest of all HIV-attributable cancers. A relatively low number of non-Hodgkin lymphoma cases in people living with HIV in Africa could be due to under-reporting as a result of inadequate diagnostic capacity.^19^ To avoid over-estimating the non-Hodgkin lymphoma PAF, we were careful to obtain age-specific RRs to apply to age-specific cancer burden (rather than applying one meta-RR to all non-Hodgkin lymphoma cases^20^, ^21^, ^22^). Receipt of ART is associated with a reduced risk of non-Hodgkin lymphoma,^23^ so we also focused on RRs in the most recent period of ART, rather than using all historical data, which would have resulted in a much higher PAF.^20^ Although the RR is expected to vary by non-Hodgkin lymphoma histology, due to the scarcity of subtype-specific data for non-Hodgkin lymphoma in GLOBOCAN 2022, we were restricted to applying the age-specific RR to the overall non-Hodgkin lymphoma burden.

Anal cancer accounted for less than 5% (3400 of 81 300) of all HIV-attributable cancer cases globally, but more than 20% in people living with HIV in North America and western and northern Europe, which is expected to be driven by the higher concentration of HIV in MSM in these regions. Indeed, the burden of HIV-attributable anal cancer in men is nearly four times higher than in women, in contrast to the burden of anal cancer in the general population, which is higher in women.^8^ Our study highlights the importance of anal cancer prevention, particularly in high-income settings. Catalysed by the ANCHOR HSIL treatment trial,^24^ as well as the accumulation of data on the epidemiology of anal cancer and screening test performance in people living with HIV, national and international guidelines are increasingly recommending anal cancer screening for people living with HIV.^25^

HIV-attributable conjunctival cancer was rarer than other HIV-attributable cancer sites, and was concentrated in sub-Saharan Africa.^9^ This is consistent with the increased risk of conjunctival cancer seen in multiple studies from sub-Saharan Africa, including South Africa, where a strong increase in conjunctival cancer incidence was observed in the pre-ART era of the HIV epidemic, and where an ecological correlation has subsequently been reported between decreasing conjunctival cancer incidence and expansion of access to ART.^26^

A small number of studies have estimated the burden of cancer cases attributable to HIV at national levels in Italy,^22^ South Korea,^27^ and the USA,^21^ although they have only focused on a smaller subset of HIV-attributable cancer sites. In 2020, 4518 (1·2%) cancer deaths in Italy were reported to be attributable to HIV, including Kaposi sarcoma, non-Hodgkin lymphoma, anal cancer, and Hodgkin lymphoma.^22^ These figures are higher than the estimates for Italy in our study for 2022 (740 [0·2%] of 405 953 cases; appendix p 11). The discrepancy is largely due to the much higher HIV prevalence estimate used in the previous study (2·0%, based on individuals visiting public outpatient clinics who reported ever undergoing HIV testing), compared to the population-based UNAIDS estimate of 0·3% used in this study. Another study from South Korea^27^ estimated that 79 cases of cancer (PAF 0·05%) were attributable to HIV in 2007, which is similar to our estimate of 27 (<0·1%) of 232 108 cases in 2022 (appendix p 12). Islami and colleagues^21^ reported that in 2014, 0·5% of all cancers among adults aged 30 years and older in the USA were attributable to HIV, which is comparable to our estimate of 3747 (0·2%) of all 1 821 719 cancer cases in people aged 15 years and older in 2022 (appendix p 15). All three of these national studies estimated a higher HIV-attributable non-Hodgkin lymphoma burden in comparison to our study, which used a more precise, age-specific, RR methodology (appendix p 7).

The primary focus of our study was to estimate the cancer burden attributable to HIV. However, our approach also allowed us to calculate the number of cancer cases in people living with HIV, irrespective of causality. When we validated our estimates of the relative distribution of cancer cases in people living with HIV against recently published reports at the country level (South Africa, Rwanda, South Korea, India, the USA, Australia, Italy, France, Switzerland, Brazil, and China; appendix pp 19–20), our findings showed broadly similar patterns to most publications. For example, in South Africa, the relative distribution of Kaposi sarcoma, non-Hodgkin lymphoma and cervical cancer was 38·9%, 15·8%, and 45·3%, respectively, among people living with HIV, versus our estimates of 32·6%, 14·3%, and 51·1%, respectively. Although we observed a tendency for a lower proportion of Kaposi sarcoma in our estimates compared with these studies based on older data, this is probably due to the reduction in its proportional importance compared to other cancer sites in recent years.

Although studies have shown increased RRs of liver, non-melanoma skin, and lung cancer in people living with HIV,^2^ we excluded these cancer sites in our analyses due to an absence of robust RRs. For liver cancer, the strongest RRs come from high-income countries where liver cancer is rare and largely caused by HCV.^12^ However, no clear association between liver cancer and HIV has been observed in Africa, where both conditions are prevalent. This could be because HBV is the leading cause of liver cancer in Africa and Asia,^28^ and ART is effective at treating both HIV and HBV.^29^ Consequently, people living with HIV receive chemoprevention for their HBV-related liver cancer risk, while HBV-positive individuals in the general population might remain undiagnosed and untreated, hence reducing the RR in people living with HIV. For both lung cancer and non-melanoma skin cancer, there is no clearly established infectious cause, and excess risks—although significant in a meta-analysis^4^—are small enough that they could potentially be the artifact of confounding risk factors (eg, smoking could confound the association between HIV infection and lung cancer).

The main strength of our analysis is that it uses the latest cancer burden data from 2022 and cancer site-specific methodology by taking into account the specific epidemiology of individual cancer types. As much as possible, relevant factors that significantly affect RRs were considered in the analysis, by stratifying data and methods by age, sex, and geography. For example, for anal cancer, countries were grouped on the basis of the expected concentration of the male HIV epidemic among MSM. Additionally, using data from GLOBOCAN enabled us to have estimates of the HIV-attributable Kaposi sarcoma burden, whereas Kaposi sarcoma cases are not included in estimates from GBD.

Limitations of our study include the fact that RR estimates can change over time, linked to ART use in people living with HIV. This is particularly relevant for Kaposi sarcoma, non-Hodgkin lymphoma, and conjunctival cancer, which are most strongly linked to the effects of ART immune reconstitution. We believe these effects were mitigated in the present analysis by assessing the PAFs for Kaposi sarcoma using data of HIV prevalence in cancer cases, the use of recent age-specific RRs of non-Hodgkin lymphoma (2013–19), and the fact that studies have shown no significant changes in RRs over time for cancers other than Kaposi sarcoma and non-Hodgkin lymphoma.^5^, ^30^ Additionally, we had no means to address confounding factors related to the common transmission of HIV and other carcinogenic infections (eg, HPV and HIV through sexual routes), potentially leading to an overestimation of HIV PAFs (but not the proportion of specific cancer cases in people living with HIV, which we validated against published data from linkage studies; appendix pp 19–20). Our estimates also assume that any existing cancer prevention initiatives, such as cervical or anal cancer screening, have equally benefited people living with HIV and the general population, which might not be the case. GLOBOCAN estimates are based on extrapolations from available cancer registry data, but many countries do not have sufficient data (about a third of countries in Africa had no cancer registry data available and their estimates were based on data from neighbouring countries). Additionally, no method was identified to reliably estimate uncertainty intervals of the PAFs, given the diverse data sources and methodologies used to calculate cancer-specific PAFs. Our estimates of HIV-attributable ASIRs represent population-level estimates for comparative purposes. Notably, we cannot estimate, nor compare, the incidence of these cancers among people living with HIV only (as done in registry linkage studies), because of the absence of data on person-years of follow-up of people living with HIV for most countries. In the absence of practical methods to address these limitations, this estimation represents the most accurate assessment achievable with the latest available data.

In conclusion, an estimated 81 300 cancer cases globally could be attributable to HIV worldwide. Our findings could inform national priorities to reduce infection-related cancers among people living with HIV, both through primary HIV control measures (eg, reducing transmission and improving ART use), and cancer-specific interventions. These priorities are underpinned by considerable geographical disparities, with a pronounced concentration of HIV-attributable cervical cancer and Kaposi sarcoma in sub-Saharan Africa, and a relatively higher burden of anal cancer and non-Hodgkin lymphoma in high-income settings.

### Contributors

### Data sharing

Data are available upon request to the corresponding author.

## Declaration of interests
