# Supplementary material for: Global burden of cancer attributable to HIV: a worldwide incidence analysis
Source: Lancet Glob Health. 2025 Aug 19;13(9):e1525–32. doi: 10.1016/S2214-109X(25)00264-5 (PMC12368413; doi:10.1016/S2214-109X(25)00264-5)
Supplement: Supplementary appendix [file mmc1.pdf]

THE LANCET
Global Health

Supplementary appendix

This appendix formed part of the original submission and has been peer reviewed. We post it as supplied by the authors.

Supplement to: Huang Y, Georges D, Rumgay H, Soerjomataram I, Clifford GM. Global burden of cancer attributable to HIV: a worldwide incidence analysis. *Lancet Glob Health* 2025; **13:** e1525–32.

Supplementary Appendix

Contents

Figure S1. Map showing overall population attributable fractions of cancer cases attributable to HIV in males and females 2
Figure S2. Map showing age-standardised incidence rates of cancers attributable to HIV in males and females 4
Figure S3. Cancers attributable to HIV across different UN regions or subregions in males and females 6
Table S1. Methodology for calculation of population attributable fraction and burden by cancer sites. 7
Table S2. Cancer cases attributable to HIV across different UN regions or subregions. 9
Table S3. The comparison of cancer diagnoses among people living with HIV between empirical data and IARC estimates 19
Reference 21

Figure S1. Map showing overall population attributable fractions of cancer cases attributable to HIV in males and females

A. Map showing overall population attributable fractions of cancer cases attributable to HIV in males

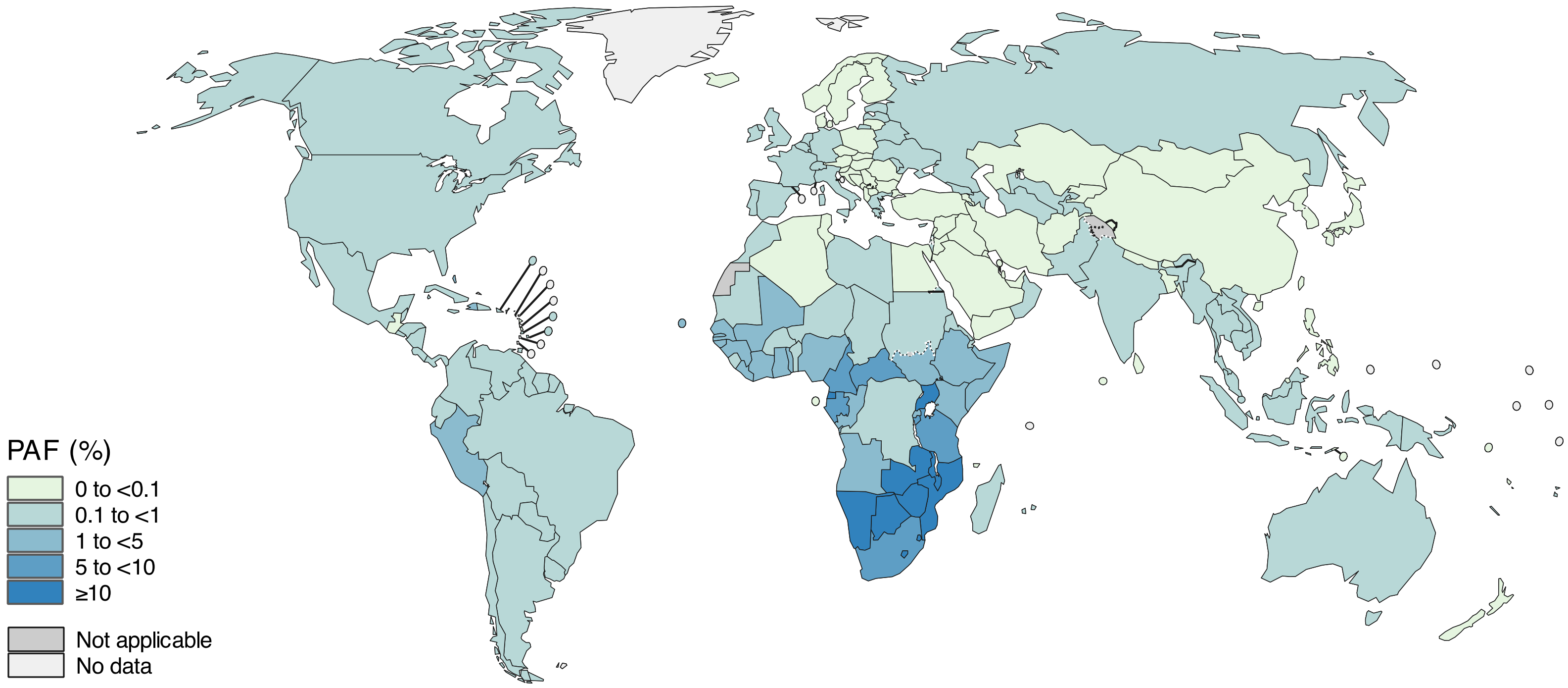

B. Map showing overall population attributable fractions of cancers attributable to HIV in females

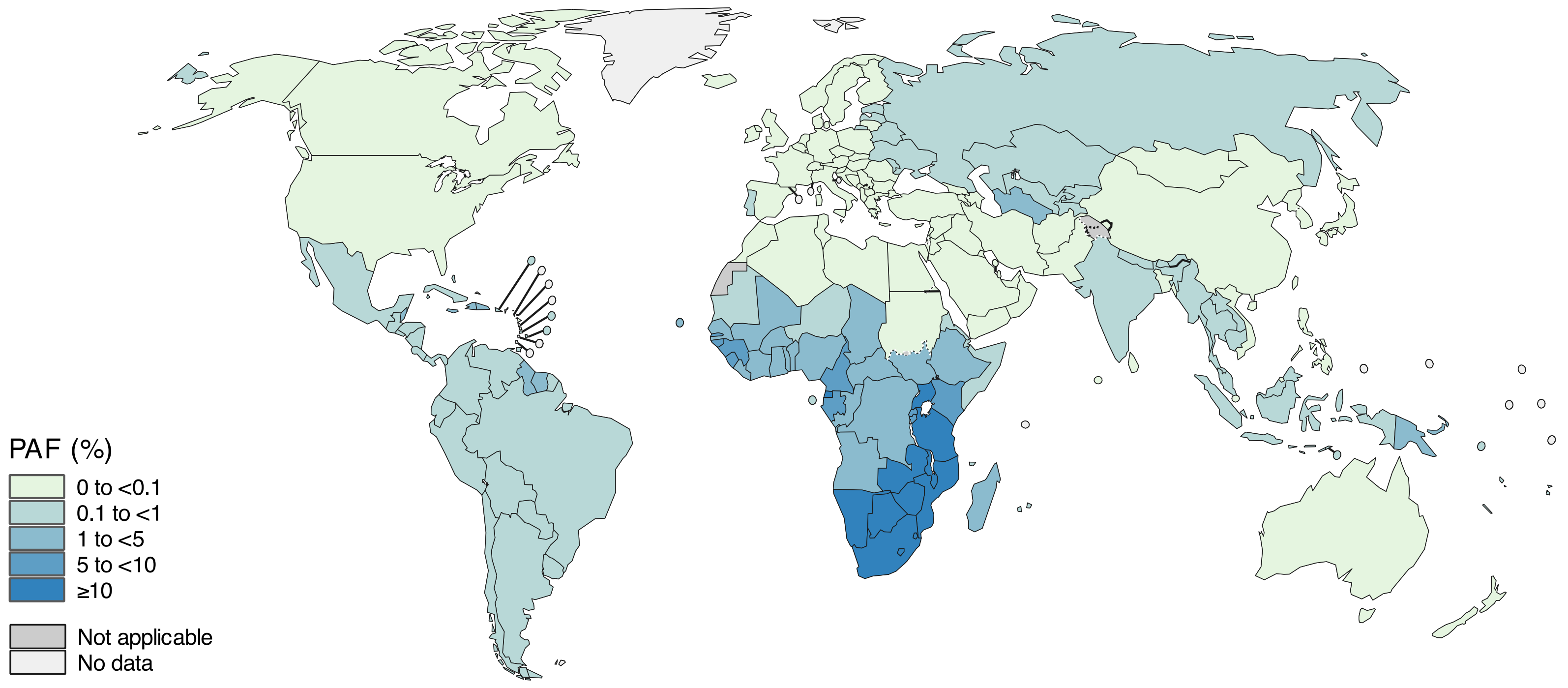

Abbreviation: PAF: population attributable fraction.

Figure S2. Map showing age-standardised incidence rates of cancers attributable to HIV in males and females

A. Map showing age-standardised incidence rates of cancers attributable to HIV in males

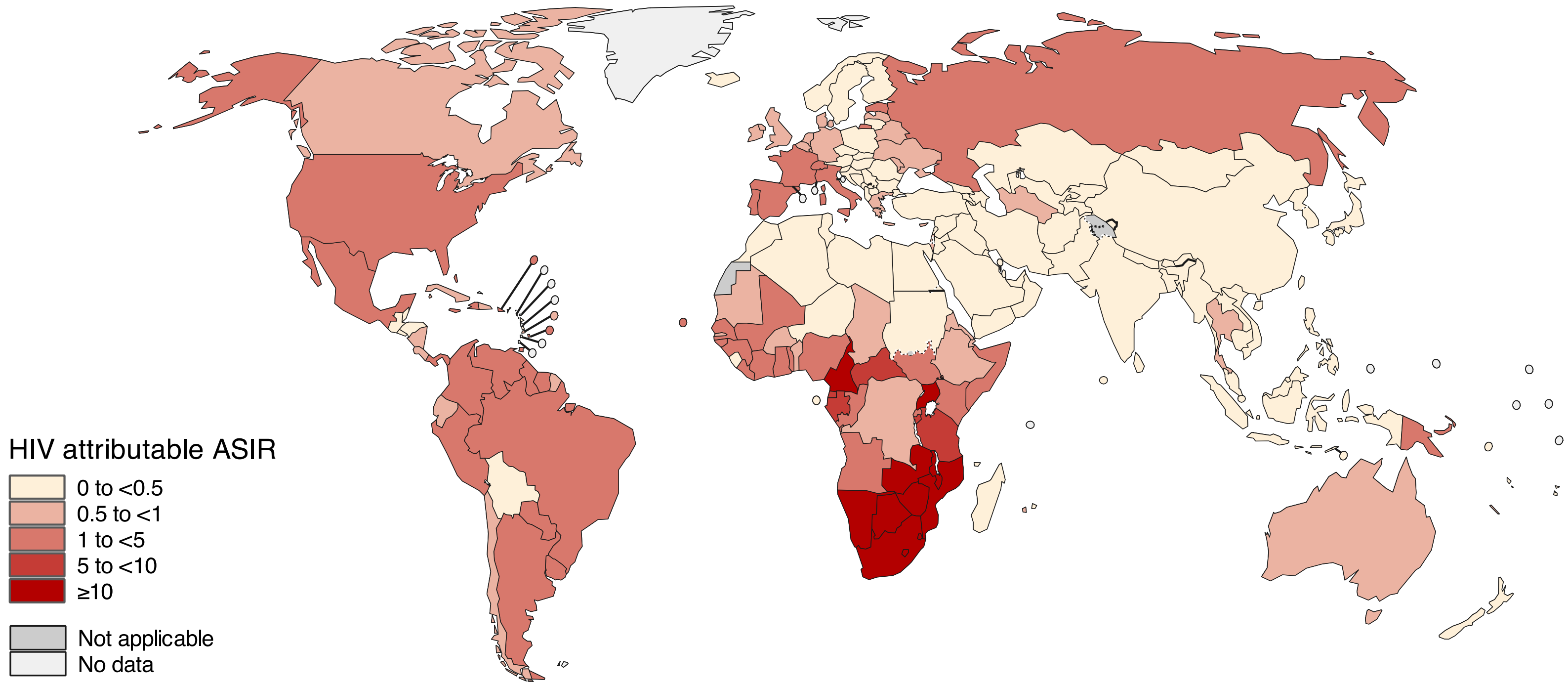

B. Map showing age-standardised incidence rates of cancers attributable to HIV in females

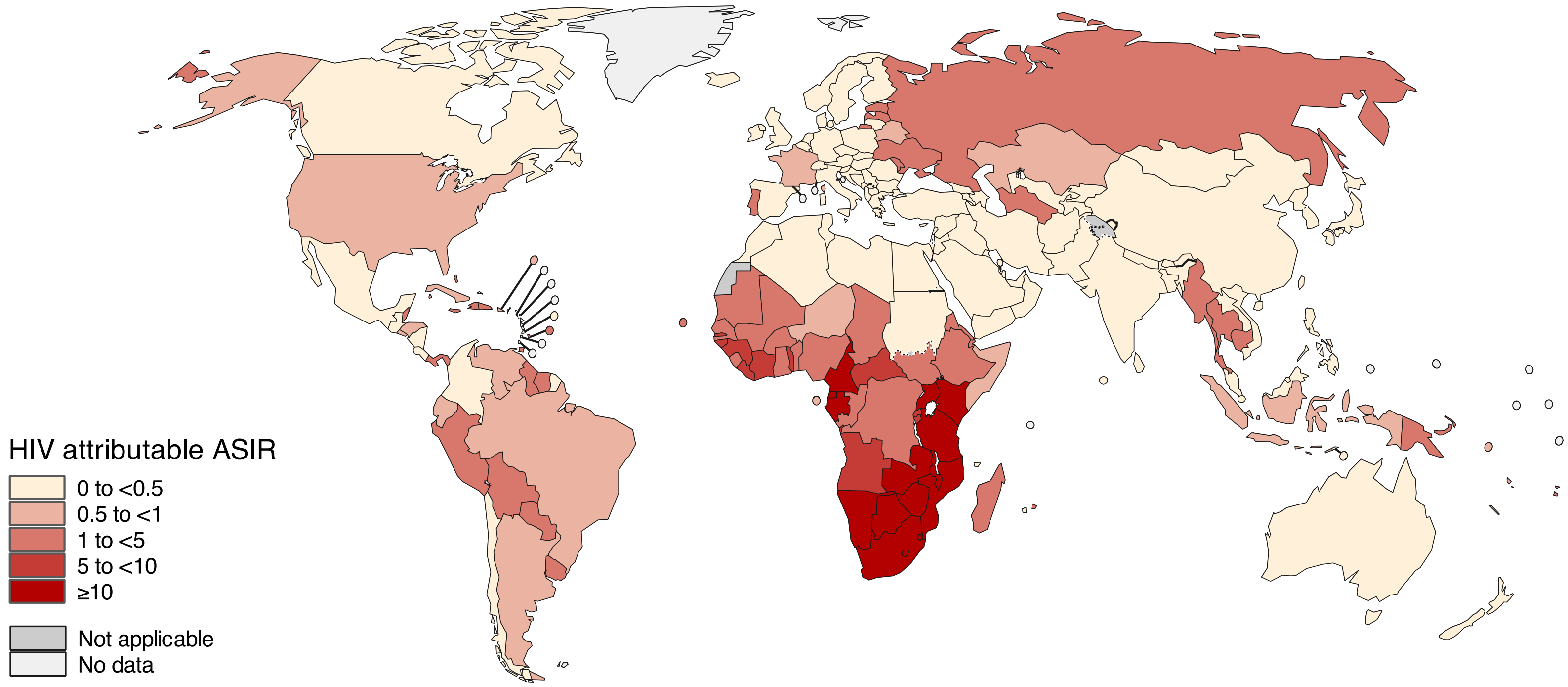

Abbreviation: ASIR: Age-standardised incidence rates.

Figure S3. Cancers attributable to HIV across different UN regions or subregions in males and females

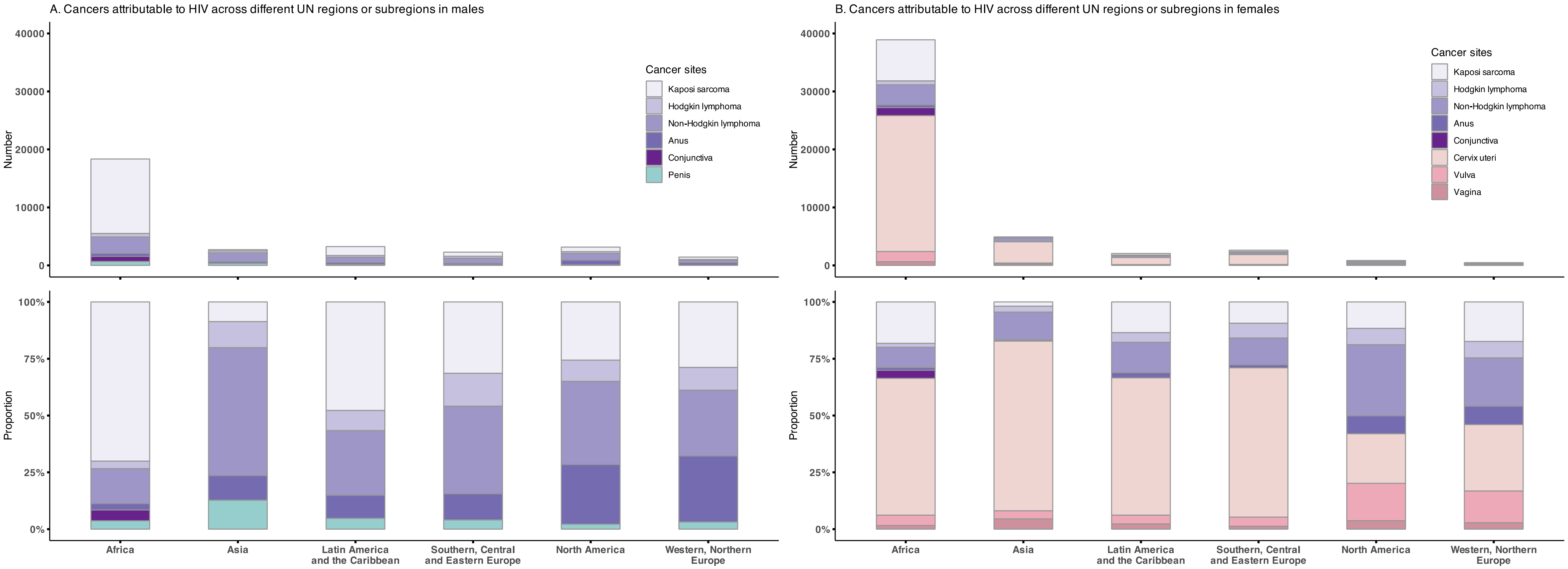

Note: The cancer cases attributable to HIV in Oceania was removed due to the small number.

Table S1. Methodology for calculation of population attributable fraction and burden by cancer sites

Cancer Sites	Methods for calculating PAF			Number of cancer cases
	Previous published methodology	Relative risk for the association of HIV with cancer site	HIV prevalence in population	
**Kaposi sarcoma**	Khalil, A.I. et al 2022[1]	Due to the very high RR for KS in PLHIV, the prevalence of HIV infection in KS cases was assumed to equate with PAF. Country and/or UN (sub)region PAFs were applied.		GLOBOCAN 2022: Country, age, and sex specific:<br>• 185 countries and territories<br>• Age groups: 5-year age-groups ranging from 15 to 80+ years<br>• Sex groups: male and/or female, depending on the cancer site<br><br>Proportions of anal squamous cell carcinoma within total anal cancer were extracted from CI5-XI registry data as previously reported[2,3], and numbers of anal squamous cell carcinoma cases were estimated by applying these proportions to the estimated numbers of anal cancer cases in GLOBOCAN.
**Cervix uteri**	Khalil, A.I. et al 2022[4,5]	Age specific RRs were applied to females only:<br>(1) 15-34 years: 33·8<br>(2) 35-44 years: 6·8<br>(3) 45-54 years: 4·2<br>(4) ≥55 years: 2·4	UNAIDS 2023; Country, age, and sex specific<br>• 172 countries and territories<br>• Age groups: 5-year age-groups ranging from 15 to 80+ years<br>• Sex groups: male and/or female, depending on the cancer site<br><br>For seven countries and territories in GLOBOCAN 2022 not included in UNAIDS dataset, HIV prevalence data were supplemented from the 2021 Global Burden of Disease.	
**Squamous cell carcinoma of the anus**	Deshmukh, A.A. et al 2023[2]	Country, age, sex specific RRs were applied:<br>(1) For females, the same age-specific RRs were applied in all countries:<br>(a) 15-59 years: 9·7<br>(b) ≥60 years: 4·3<br>(2) For males, the age-specific RRs were applied to countries classified in three categories according to the relative burden of HIV infection among men versus women (as a surrogate for the concentration of the male HIV epidemic in MSM):<br>(a) Countries with >70% of HIV burden among men (predominantly MSM):<br>15-59 years: 121·7<br>≥60 years: 36·0<br>(b) 50% to 70% (MSM and MSW):<br>15-59 years: 82·0<br>≥60 years: 32·0<br>(c) <50% (predominantly MSW):<br>15-59 years: 36·6<br>≥60 years: 29·3		
**Non-Hodgkin lymphoma**		Age specific SIRs were applied, irrespective of sex, from the period 2013-2019 from the U.S. HIV AIDS CANCER Match Study[6]:<br>(1) 15-39 years: 18·9<br>(2) 40-59 years: 7·8<br>(3) ≥60 years: 2·5		
**Hodgkin lymphoma**		10·9, applied irrespective of sex[7]		

**Penis**		7·6, applied to males only[7]		
**Vulva**		14·1, applied to females only[7]		
**Vagina**		14·1, applied to females only[7]		
**Squamous cell carcinoma of the conjunctiva**	Hammerl, L. et al 2019	10·0, applied to Africa only, irrespective of sex[8]		The number of conjunctiva cancer cases in each Africa country was generated by applying the age-specific incidence, that was estimated based on previous IARC methodology using data from the African Cancer Registry network, to 2022 population figures. The small burden in regions outside Africa (n<50) was not considered HIV attributable.

Abbreviations: RR, relative risk; SIR, standardized incidence ratio; PAF, population attributable fraction; PLHIV, people living with HIV; MSM, men who have sex with men; MSW, men who have sex with women.

Table S2. Cancer cases attributable to HIV by countries.

ISO3	Country or territory	Cancer cases attributable to HIV	PAF (%)	ASIR (per 100,000)
AFG	Afghanistan	12	<0.1	<0.1
AGO	Angola	910	4.3	5.4
ALB	Albania	4	<0.1	0.1
ARE	United Arab Emirates	1	<0.1	<0.1
ARG	Argentina	364	0.3	1.0
ARM	Armenia	5	<0.1	0.2
AUS	Australia	122	<0.1	0.5
AUT	Austria	23	<0.1	0.2
AZE	Azerbaijan	18	0.1	0.2
BDI	Burundi	521	7.0	8.8
BEL	Belgium	84	0.1	0.7
BEN	Benin	79	1.1	1.3
BFA	Burkina Faso	162	1.2	1.6
BGD	Bangladesh	12	<0.1	<0.1
BGR	Bulgaria	10	<0.1	0.1
BHR	Bahrain	0	<0.1	<0.1
BHS	Bahamas	10	1.1	3.0
BIH	Bosnia and Herzegovina	1	<0.1	<0.1
BLR	Belarus	61	0.1	0.7
BLZ	Belize	3	0.9	1.1
BOL	Bolivia, Plurinational State of	59	0.4	0.7
BRA	Brazil	1,623	0.3	0.9
BRB	Barbados	8	0.7	3.5
BRN	Brunei	1	<0.1	0.2
BTN	Bhutan	1	0.1	0.1
BWA	Botswana	641	29.5	39.2

CAF	Central African Republic	120	4.7	6.1
CAN	Canada	252	0.1	0.6
CHE	Switzerland	63	0.1	0.6
CHL	Chile	106	0.2	0.6
CHN	China	1,356	<0.1	0.1
CIV	Côte d'Ivoire	692	3.4	5.5
CMR	Cameroon	1,538	8.4	11.9
COD	Congo, Democratic Republic of	727	1.5	1.6
COG	Congo, Republic of	79	3.0	2.7
COL	Colombia	540	0.5	1.2
COM	Comoros	0	<0.1	<0.1
CPV	Cabo Verde	8	1.9	1.9
CRI	Costa Rica	30	0.3	0.6
CUB	Cuba	77	0.2	0.8
CYP	Cyprus	5	<0.1	0.4
CZE	Czechia	15	<0.1	0.1
DEU	Germany	381	<0.1	0.4
DJI	Djibouti	6	0.8	0.9
DNK	Denmark	26	<0.1	0.4
DOM	Dominican Republic	135	0.7	1.6
DZA	Algeria	32	<0.1	<0.1
ECU	Ecuador	106	0.4	0.8
EGY	Egypt	47	<0.1	<0.1
ERI	Eritrea	17	0.8	1.0
ESP	Spain	479	0.2	0.9
EST	Estonia	16	0.2	1.3
ETH	Ethiopia	1,100	1.5	1.9
FIN	Finland	17	<0.1	0.2
FJI	Fiji	7	0.4	1.0

FRA	France	656	0.2	1.0
GAB	Gabon	117	6.5	8.2
GBR	United Kingdom	430	0.1	0.7
GEO	Georgia	12	<0.1	0.4
GHA	Ghana	603	2.3	3.1
GIN	Guinea	408	4.9	5.4
GLP	France, Guadeloupe	1	<0.1	0.4
GMB	The Republic of the Gambia	63	5.5	4.3
GNB	Guinea-Bissau	49	4.5	4.4
GNQ	Equatorial Guinea	100	11.5	12.3
GRC	Greece	57	<0.1	0.4
GTM	Guatemala	33	0.2	0.3
GUF	French Guyana	1	0.2	0.6
GUM	Guam	0	<0.1	<0.1
GUY	Guyana	15	1.3	2.6
HND	Honduras	35	0.4	0.6
HRV	Croatia	8	<0.1	0.1
HTI	Haiti	179	1.4	2.4
HUN	Hungary	11	<0.1	0.1
IDN	Indonesia	1,069	0.3	0.5
IND	India	2,842	0.2	0.3
IRL	Ireland	24	<0.1	0.5
IRN	Iran, Islamic Republic of	41	<0.1	<0.1
IRQ	Iraq	7	<0.1	<0.1
ISL	Iceland	1	<0.1	0.2
ISR	Israel	26	<0.1	0.4
ITA	Italy	740	0.2	0.9
JAM	Jamaica	63	0.9	2.6
JOR	Jordan	2	<0.1	<0.1

JPN	Japan	60	<0.1	<0.1
KAZ	Kazakhstan	53	0.2	0.3
KEN	Kenya	2,768	6.5	9.6
KGZ	Kyrgyzstan	12	0.2	0.2
KHM	Cambodia	77	0.4	0.7
KOR	Korea, Republic of	27	<0.1	<0.1
KWT	Kuwait	1	<0.1	<0.1
LAO	Lao People's Democratic Republic	19	0.2	0.3
LBN	Lebanon	6	<0.1	0.1
LBR	Liberia	99	2.7	3.3
LBY	Libya	9	0.1	0.2
LCA	Saint Lucia	1	0.2	0.4
LKA	Sri Lanka	3	<0.1	<0.1
LSO	Lesotho	539	27.8	38.6
LTU	Lithuania	11	<0.1	0.3
LUX	Luxembourg	3	<0.1	0.5
LVA	Latvia	15	0.1	1.0
MAR	Morocco	64	0.1	0.2
MDA	Republic of Moldova	42	0.3	1.1
MDG	Madagascar	270	1.4	1.4
MDV	Maldives	0	<0.1	<0.1
MEX	Mexico	794	0.4	0.8
MKD	The former Yugoslav Republic of Macedonia	1	<0.1	<0.1
MLI	Mali	321	2.3	3.2
MLT	Malta	4	0.1	0.7
MMR	Myanmar	428	0.6	1.0
MNE	Montenegro	1	<0.1	0.1
MNG	Mongolia	0	<0.1	<0.1
MOZ	Mozambique	6,871	28.5	39.5

MRT	Mauritania	30	1.0	1.1
MTQ	France, Martinique	1	<0.1	0.3
MUS	Mauritius	10	0.4	0.9
MWI	Malawi	4,511	25.5	42.9
MYS	Malaysia	80	0.2	0.3
NAM	Namibia	592	18.8	38.0
NCL	France, New Caledonia	1	0.1	0.5
NER	Niger	48	0.5	0.4
NGA	Nigeria	2,587	2.2	2.8
NIC	Nicaragua	27	0.3	0.5
NLD	The Netherlands	98	<0.1	0.6
NOR	Norway	19	<0.1	0.3
NPL	Nepal	28	0.1	0.1
NZL	New Zealand	10	<0.1	0.2
OMN	Oman	5	0.1	0.1
PAK	Pakistan	189	0.1	0.1
PAN	Panama	63	0.8	1.8
PER	Peru	503	0.7	1.8
PHL	Philippines	78	<0.1	<0.1
PNG	Papua New Guinea	172	1.5	2.8
POL	Poland	49	<0.1	0.1
PRI	Puerto Rico	22	0.2	0.9
PRK	Korea, Democratic Republic of	17	<0.1	<0.1
PRT	Portugal	179	0.3	1.8
PRY	Paraguay	94	0.7	1.7
PSE	Gaza Strip and West Bank	1	<0.1	<0.1
PYF	French Polynesia	1	0.1	0.4
QAT	Qatar	1	<0.1	<0.1
REU	France, La Réunion	4	0.1	0.5

ROU	Romania	52	<0.1	0.3
RUS	Russian Federation	2,729	0.4	2.4
RWA	Rwanda	393	6.0	5.7
SAU	Saudi Arabia	16	<0.1	<0.1
SDN	Sudan	28	0.1	0.1
SEN	Senegal	138	1.2	1.6
SGP	Singapore	14	<0.1	0.2
SLB	Solomon Islands	2	0.3	0.4
SLE	Sierra Leone	73	4.0	1.3
SLV	El Salvador	24	0.3	0.5
SOM	Somalia	68	0.7	1.0
SRB	Serbia	12	<0.1	0.1
SSD	South Sudan	260	4.0	4.5
STP	Sao Tome and Principe	1	0.4	0.5
SUR	Suriname	14	1.3	3.0
SVK	Slovakia	5	<0.1	<0.1
SVN	Slovenia	2	<0.1	<0.1
SWE	Sweden	35	<0.1	0.3
SWZ	Eswatini	411	39.3	62.6
SYR	Syrian Arab Republic	2	<0.1	<0.1
TCD	Chad	171	1.8	2.3
TGO	Togo	164	3.2	3.6
THA	Thailand	681	0.4	1.1
TJK	Tajikistan	12	0.2	0.2
TKM	Turkmenistan	57	0.9	1.2
TLS	Timor-Leste	2	0.2	0.2
TTO	Trinidad and Tobago	28	0.7	2.3
TUN	Tunisia	10	<0.1	<0.1
TUR	Turkey	71	<0.1	<0.1

TZA	Tanzania, United Republic of	3,788	9.0	13.6
UGA	Uganda	6,401	19.3	28.2
UKR	Ukraine	440	0.3	1.2
URY	Uruguay	42	0.3	1.5
USA	United States of America	3,747	0.2	1.2
UZB	Uzbekistan	96	0.3	0.4
VEN	Venezuela, Bolivarian Republic of	293	0.5	1.3
VNM	Viet Nam	184	0.1	0.2
VUT	Vanuatu	1	0.3	0.4
WSM	Samoa	0	<0.1	0.3
YEM	Yemen	7	<0.1	<0.1
ZAF	South Africa	11,772	11.8	25.8
ZMB	Zambia	3,500	24.5	39.5
ZWE	Zimbabwe	3,339	19.8	44.1

Table S3. Cancer cases attributable to HIV across different UN regions or subregions.

Cancer sites	N (proportion*, %)
Africa	
Kaposi sarcoma	20,000(34.9)
Hodgkin lymphoma	1,300(2.3)
Non-Hodgkin lymphoma	6,300(11)
Squamous cell carcinoma of the anus	1,000(1.7)
Conjunctiva	2,300(4)
Penis	700(1.2)
Cervix uteri	23,400(40.8)
Vulva	1,800(3.1)
Vagina	600(1.0)
Asia	
Kaposi sarcoma	300(3.9)
Hodgkin lymphoma	400(5.3)
Non-Hodgkin lymphoma	2,100(27.6)
Squamous cell carcinoma of the anus	300(3.9)
Conjunctiva	NA
Penis	300(3.9)
Cervix uteri	3,700(48.7)
Vulva	200(2.6)
Vagina	200(2.6)
Latin America and the Caribbean	
Kaposi sarcoma	1,800(34)
Hodgkin lymphoma	400(7.5)
Non-Hodgkin lymphoma	1,200(22.6)
Squamous cell carcinoma of the anus	400(7.5)

Conjunctiva	NA
Penis	200(3.8)
Cervix uteri	1,200(22.6)
Vulva	<100(1.5)
Vagina	<100(0.9)
Southern, Central and Eastern Europe	
Kaposi sarcoma	1,000(20.4)
Hodgkin lymphoma	500(10.2)
Non-Hodgkin lymphoma	1,200(24.5)
Squamous cell carcinoma of the anus	300(6.1)
Conjunctiva	NA
Penis	<100(1.9)
Cervix uteri	1,700(34.7)
Vulva	100(2.0)
Vagina	<100(0.7)
North America	
Kaposi sarcoma	900(22.5)
Hodgkin lymphoma	400(10.0)
Non-Hodgkin lymphoma	1,400(35.0)
Squamous cell carcinoma of the anus	900(22.5)
Conjunctiva	NA
Penis	<100(1.7)
Cervix uteri	200(5.0)
Vulva	100(2.5)
Vagina	<100(0.8)
Western, Northern Europe	
Kaposi sarcoma	500(26.3)
Hodgkin lymphoma	200(10.5)

Non-Hodgkin lymphoma	500(26.3)
Squamous cell carcinoma of the anus	500(26.3)
Conjunctiva	NA
Penis	<100(2.4)
Cervix uteri	100(5.3)
Vulva	<100(3.5)
Vagina	<100(0.7)
Oceania	
Kaposi sarcoma	<100(14.0)
Hodgkin lymphoma	<100(5.0)
Non-Hodgkin lymphoma	<100(28.3)
Squamous cell carcinoma of the anus	<100(13.7)
Conjunctiva	NA
Penis	<100(1.3)
Cervix uteri	100(33.3)
Vulva	<100(1.7)
Vagina	<100(1.3)

Note: * For small case counts (<100), proportions were calculated based on numbers before rounding to the nearest hundred.

Table S4. The comparison of the distribution of cancer diagnoses among people living with HIV between empirical data and IARC estimates

Country	Empirical data					IARC estimates, 2022
	Reference	Year followed up	Cancer sites	Case number	Proportion out of all HIV-positive cancer cases (%)	Proportion out of all HIV-attributable cancer cases (%)
Rwanda	Dusingize et al., 2024[9]	2007-2018	Kaposi sarcoma	250	40·8	29·4
			Non-Hodgkin's lymphoma	89	14·5	17·5
			Cervical cancer	264	43·1	52·0
			Anus	10	1·6	1·2
Republic of Korea	Park et al., 2022[10]	2006-2018	Kaposi sarcoma	20	16·7	15·3
			Non-Hodgkin lymphoma	73	60·8	61·9
			Cervix uteri	7	5·8	10·4
			Anus	20	16·7	12·3
South Africa	Muchengeti et al., 2022[11]	2004–2014	Kaposi sarcoma	6373	38·9	32·6
			Non-Hodgkin lymphoma	2588	15·8	14·3
			Cervix uteri	7433	45·3	51·1
			Anus	0	0·0	2·0
Australia	Wong et al., 2022[12]	2009-2012	Kaposi sarcoma	65	35·8	30·9
			Lymphoma(HL & NHL)	71	39·3	39·2
			Cervical	0	0·0	2·4
			Anus	45	24·9	27·6
France	Poizot-Martin et al., 2021[13]	2010-2015	Kaposi sarcoma	137	34·9	22·1
			Non-Hodgkin lymphoma	182	46·4	40·9
			Cervix uteri	16	4·1	9·1
			Anus	57	14·5	28·0

China	Zhu et al., 2019[14]	2008-2011	Kaposi sarcoma	171	23·8	10·0
			Lymphoma(NL & NHL)	416	58·0	37·5
			Cervix uteri	128	17·9	51·0
			Anus	2	0·3	1·5
Brazil	Tanaka et al., 2018[15]	1997-2012	Kaposi sarcoma	503	46·1	26·6
			Non-Hodgkin lymphoma	400	36·7	26·8
			Cervix uteri	114	10·5	31·2
			Anus	73	6·7	15·4
The United States	Hernández-Ramírez et al, 2017[16]	1996-2012	Kaposi sarcoma	2,269	28·5	23·0
			Non-Hodgkin lymphoma	3,687	46·4	46·6
			Cervix uteri	428	5·4	5·8
			Anus	1,568	19·7	24·6
India	Godbole et al., 2016[17]	1991-2009	Kaposi sarcoma	0	0·0	0·2
			Non-Hodgkin lymphoma	15	30·6	29·5
			Cervix uteri	34	69·4	70·3
			Anus	-	-	-
Italy	Calabresi et al., 2013[18]		Kaposi sarcoma	96	46·8	52·5
			Non-Hodgkin lymphoma	95	46·3	32·2
			Cervix uteri	9	4·4	3·0
			Anus	5	2·4	12·3
Switzerland	Franceschi et al., 2010[19]	2002 – 2006	Kaposi sarcoma	14	26·9	34·4
			Non-Hodgkin lymphoma	32	61·5	34·8
			Cervix uteri	0	0·0	3·8
			Anus	6	11·5	27·0

Reference

1. Ibrahim Khalil A, Franceschi S, de Martel C, Bray F, Clifford GM. Burden of Kaposi sarcoma according to HIV status: A systematic review and global analysis. *Int J Cancer* 2022; **150**(12): 1948-57.
2. Deshmukh AA, Damgacioglu H, Georges D, et al. Global burden of HPV-attributable squamous cell carcinoma of the anus in 2020, according to sex and HIV status: A worldwide analysis. *Int J Cancer* 2023; **152**(3): 417-28.
3. de Martel C, Georges D, Bray F, Ferlay J, Clifford GM. Global burden of cancer attributable to infections in 2018: a worldwide incidence analysis. *Lancet Glob Health* 2020; **8**(2): e180-e90.
4. Ibrahim Khalil A, Mpunga T, Wei F, et al. Age-specific burden of cervical cancer associated with HIV: A global analysis with a focus on sub-Saharan Africa. *Int J Cancer* 2022; **150**(5): 761-72.
5. Mpunga T, Znaor A, Uwizeye FR, et al. A case-control study of HIV infection and cancer in the era of antiretroviral therapy in Rwanda. *Int J Cancer* 2018; **143**(6): 1348-55.
6. HIV/AIDS Cancer Match Study. https://dceg.cancer.gov/research/who-we-study/cohorts/hiv-aids-cancer-match-study (accessed May 28 2025).
7. Yuan T, Hu Y, Zhou X, et al. Incidence and mortality of non-AIDS-defining cancers among people living with HIV: A systematic review and meta-analysis. *EClinicalMedicine* 2022; **52**: 101613.
8. International Agency for Research on Cancer. Biological Agents, IARC Monographs on the Evaluation of Carcinogenic Risks to Humans Volume 100B. https://publications.iarc.fr/Book-And-Report-Series/Iarc-Monographs-On-The-Identification-Of-Carcinogenic-Hazards-To-Humans/Biological-Agents-2012 (accessed May 28 2025).
9. Dusingize JC, Murenzi G, Muhoza B, et al. Cancer risk among people living with Human Immuodeficiency Virus (HIV) in Rwanda from 2007 to 2018. *Int J Cancer* 2024.
10. Park B, Ahn KH, Choi Y, et al. Cancer Incidence Among Adults With HIV in a Population-Based Cohort in Korea. *JAMA Netw Open* 2022; **5**(8): e2224897.
11. Muchengeti M, Bartels L, Olago V, et al. Cohort profile: the South African HIV Cancer Match (SAM) Study, a national population-based cohort. *BMJ Open* 2022; **12**(4): e053460.
12. Wong IKJ, Grulich AE, Poynten IM, et al. Time trends in cancer incidence in Australian people living with HIV between 1982 and 2012. *HIV Med* 2022; **23**(2): 134-45.
13. Poizot-Martin I, Lions C, Allavena C, et al. Spectrum and Incidence Trends of AIDS- and Non-AIDS-Defining Cancers between 2010 and 2015 in the French Dat'AIDS Cohort. *Cancer Epidemiol Biomarkers Prev* 2021; **30**(3): 554-63.
14. Zhu W, Mao Y, Tang H, et al. Spectrum of malignancies among the population of adults living with HIV infection in China: A nationwide follow-up study, 2008-2011. *PLoS One* 2019; **14**(7): e0219766.
15. Tanaka LF, Latorre M, Gutierrez EB, et al. Risk for cancer among people living with AIDS, 1997-2012: the Sao Paulo AIDS-cancer linkage study. *Eur J Cancer Prev* 2018; **27**(4): 411-7.
16. Hernandez-Ramirez RU, Shiels MS, Dubrow R, Engels EA. Cancer risk in HIV-infected people in the USA from 1996 to 2012: a population-based, registry-linkage study. *Lancet HIV* 2017; **4**(11): e495-e504.
17. Godbole SV, Nandy K, Gauniyal M, et al. HIV and cancer registry linkage identifies a substantial burden of cancers in persons with HIV in India. *Medicine (Baltimore)* 2016; **95**(37): e4850.
18. Calabresi A, Ferraresi A, Festa A, et al. Incidence of AIDS-defining cancers and virus-related and non-virus-related non-AIDS-defining cancers among HIV-infected patients compared with the general population in a large health district of Northern Italy, 1999-2009. *HIV Med* 2013; **14**(8): 481-90.

19. Franceschi S, Lise M, Clifford GM, et al. Changing patterns of cancer incidence in the early- and late-HAART periods: the Swiss HIV Cohort Study. *Br J Cancer* 2010; **103**(3): 416-22.
